# Imputation methods for missing failure times in recurrent-event survival analysis: Application to suicide attempts in the transgender population

**DOI:** 10.1371/journal.pone.0278913

**Published:** 2022-12-09

**Authors:** Shanshan Liu, Sari L. Reisner, Jody L. Herman, Edie Weller

**Affiliations:** 1 Biostatistics and Research Design Center, Institutional Centers for Clinical and Translational Research, Boston Children’s Hospital, Boston, Massachusetts, United States of America; 2 Division of Endocrinology, Diabetes and Hypertension, Brigham and Women’s Hospital, Boston, Massachusetts, United States of America; 3 Department of Medicine, Harvard Medical School, Boston, Massachusetts, United States of America; 4 Department of Epidemiology, Harvard T.H. Chan School of Public Health, Boston, Massachusetts, United States of America; 5 The Fenway Institute, Fenway Health, Boston, Massachusetts, United States of America; 6 Williams Institute, School of Law, University of California, Los Angeles, Los Angeles, California, United States of America; 7 Department of Pediatrics, Harvard Medical School, Boston, Massachusetts, United States of America; Iowa State University, UNITED STATES

## Abstract

Suicide risk among transgender populations is an important public health issue. In a project evaluating association between gender affirmation and suicide attempts in the US Transgender Survey, we evaluated the relationship between gender affirmation and risk for suicide attempts. One of the challenges is that the age at suicide attempts was only collected for the first and last attempt. The initial zero-inflated negative binomial model enabled us to evaluate the association between gender affirmation and number of suicide attempts per 5 years adjusting for other covariates. However, ignoring missing failure times of recurrent events may have caused bias and loss of efficiency. In this paper, we use a recurrent-event survival analysis incorporating time-varying covariates with three approaches to impute the age at suicide attempt, estimates from three imputation approaches are similar. We were able to confirm the findings from the initial model and identify additional associations that were not detected in the initial analysis. Findings suggest the need to consider additional analytical approaches in settings with high data missingness by design. Research to validate and compare measures that ask first and last attempt to those which enumerate all attempts in this population will be important for future surveys.

## Introduction

Suicide risk among transgender youth and adult populations is an important public health issue [1–3]. Transgender individuals (those who have a gender identity that differs from their sex assigned at birth) have a two- to four-fold increased risk of suicide attempt compared to cisgender (non-transgender) individuals [4–6]. Identifying culturally-specific factors associated with suicide risk in vulnerable populations is important to inform national prevention strategies [7]. Gender affirmation is one such transgender-specific factor to consider in suicide risk. Gender affirmation refers to changing one’s gender presentation to affirm or reflect one’s internal gender identity [8]. It includes social affirmation (socially presenting in one’s identified gender identity) such as using a selected name and pronoun, and medical affirmation (interventions or treatments that physically alter the body) such as hormones or surgery [9].

In 2015, the US Transgender Survey [10] was conducted to learn about transgender experiences on a variety of health topics, including suicide risk. A question of interest is whether there is a relationship between a gender affirmation and risk for suicide attempts. One part of the survey asked participants about suicide attempts. These questions provide information on the date of the first attempt and the date of the last attempt as well the number of attempts in between these two dates. The date of the attempts between the first and last date were not collected. If the times of each attempt were collected, a recurrent survival analysis could be implemented.

Due to the missing information on timing of each suicide attempt, the initial analysis [11, 12] used a weighted time-varying survival analysis to evaluate the association of gender affirmation with time to first suicide and a zero-inflated negative binomial model with a 5-year offset and over-dispersion parameter to assess the association of suicide attempt rate over lifetime with gender affirmation. Both models were stratified by age at survey completion and were adjusted for other factors such as race, education, and age at transgender awareness.

While these approaches identified important associations, ignoring missing failure times of recurrent events may have caused bias and loss of efficiency. In the survival analysis of the first attempt, age at the first suicide and gender affirmation (time-varying covariate) were analyzed while information about the total number of attempts and age at the last attempt was discarded. Whereas, in the analysis of total number of attempts using a zero-inflated negative binomial model, number of attempts offset by total years of follow-up and gender affirmation as static variables were analyzed, while information on timing of attempts and gender affirmation was discarded, causing lack of temporality between exposures and outcome. To overcome the shortcomings in both analyses, it is important to consider alternative approaches that would incorporate all the information on exposure and outcome simultaneously.

The missing suicide attempt dates can be viewed as a missing data problem. In this case the missing data provide information on the timing of the outcome. Missing data is a common challenge in research studies and a large body of literature exists addressing the statistical issues that arise [13–15]. It is well recognized that missing data results in bias and efficiency loss and several methods have been proposed to address this issue [16–20]. Multiple imputation is one of the approaches that is often used to address this problem. Briefly, multiple imputation involves generating multiple datasets by replacing the missing values in the sample data with plausible draws generated from the predictive distribution of models fit on the observed data. One of the assumptions to implement multiple imputation is that the data are missing completely at random (MCAR) or at least missing at random (MAR) [13]. The data are said to be MCAR if the probability of being missing is the same for all cases. The data are said to be MAR if missingness is systematically related to the observed data but not the unobserved [21]. Given that the missingness in this study is by design, it does not depend on the values would have been observed. Thus, it is reasonable to assume that these data are at least missing at random and therefore, conventional imputation methods can be utilized. For the subjects with more than two suicide attempts we do not have the age of attempt, but we do know this age fell between the age at the first and the last attempt. If we had the exact age of every suicide attempt, we could utilize recurrent survival analysis methods. In this paper, we implement three imputation approaches utilizing the age distribution of the suicide attempt and compare the results of recurrent survival analysis for the three approaches as well as to the results from the initial zero-inflated negative binomial model that did not involve missing data imputation. If the results are different this could result in some recommendations for modifying the study design for future surveys.

## Method

### Study population

The 2015 U.S. Transgender Survey (USTS) is the largest survey targeted at the transgender adults residing in the United States. Details on survey methodology are published elsewhere [11]. “transgender” was defined broadly for this study to include any individuals who identify as transgender, trans, genderqueer, non-binary, and other identities on the transgender identity spectrum. The web-based survey had 32 sections and 324 possible questions that covered various topics. The study sample included 27,715 respondents for whom data were collected over a 34-day period in the summer of 2015, among which 24,343 reported wanting or having had surgery or hormones. There were 4561 participants excluded because a) age of transgender awareness or age at first attempt was missing or b) age of transgender awareness was after date of suicide attempt or date of gender affirmation, or last attempt. Among the remaining 19,782, there are 14,231 participants who identify as binary. Analyses were a priori restricted to binary-identified participants (i.e., trans men, trans women) because gender affirmation needs and practices differ for this group relative to non-binary individuals (i.e., genderqueer, genderfluid) [22].

Written informed consent was obtained for all subjects included in this study. The survey used informed consent with a waiver of signature to protect anonymity of survey subjects. The participants were given a study info sheet at the beginning of the survey and were asked if they agreed and consented to take the survey before proceeding into the survey. The survey was reviewed and approved by the UCLA North General IRB.

### Important covariates

Various covariates are used in the analysis including gender affirmation, age at survey completion (current age), age at transgender awareness, race, ethnicity, assigned sex at birth, and education [11].

Three types of gender affirmation processes are of interest: social affirmation, hormones, and surgery. While the survey captures age at the first uptake of each gender affirmation process, it does not provide information on dosage and types of hormones, or number and types of surgical procedures received over gender time. Thus, it is difficult to study dose-response precisely. Based on age at the first use of each type of gender affirmation, we derived three time-varying binary variables indicating whether the participant had certain gender affirmation at a specific time (denoted as t). Age at survey completion was grouped into four categories: 18–24, 25–29, 30–39, and 40 or above. Age at transgender awareness (first recognizing oneself as being transgender) was also grouped into four categories: 10 or below, 10–14 (including 14), 14–18 (including 18), and above 18. We categorized these two age variables differently because awareness usually occurs at an early age. Race questions were drawn from the American Community Survey (ACS) and collapsed into 6 categories: Alaska Native/American Indian alone, Asian/Native Hawaiians (NH)/Pacific Islanders (PI), Biracial/Multiracial/Not listed, Black/African American alone, Latino/a/Hispanic alone, and White/Middle Eastern (ME)/ North African (NA) alone. Sex assigned at birth (male or female) was included as a binary variable. The highest level of education was recorded into 6 categories based on ACS: less than high school, high school graduate (including GED), some college (no degree), associate’s degree, bachelor’s degree, and graduate or professional degree.

### Assessment of suicide attempts

**Fig 1:**
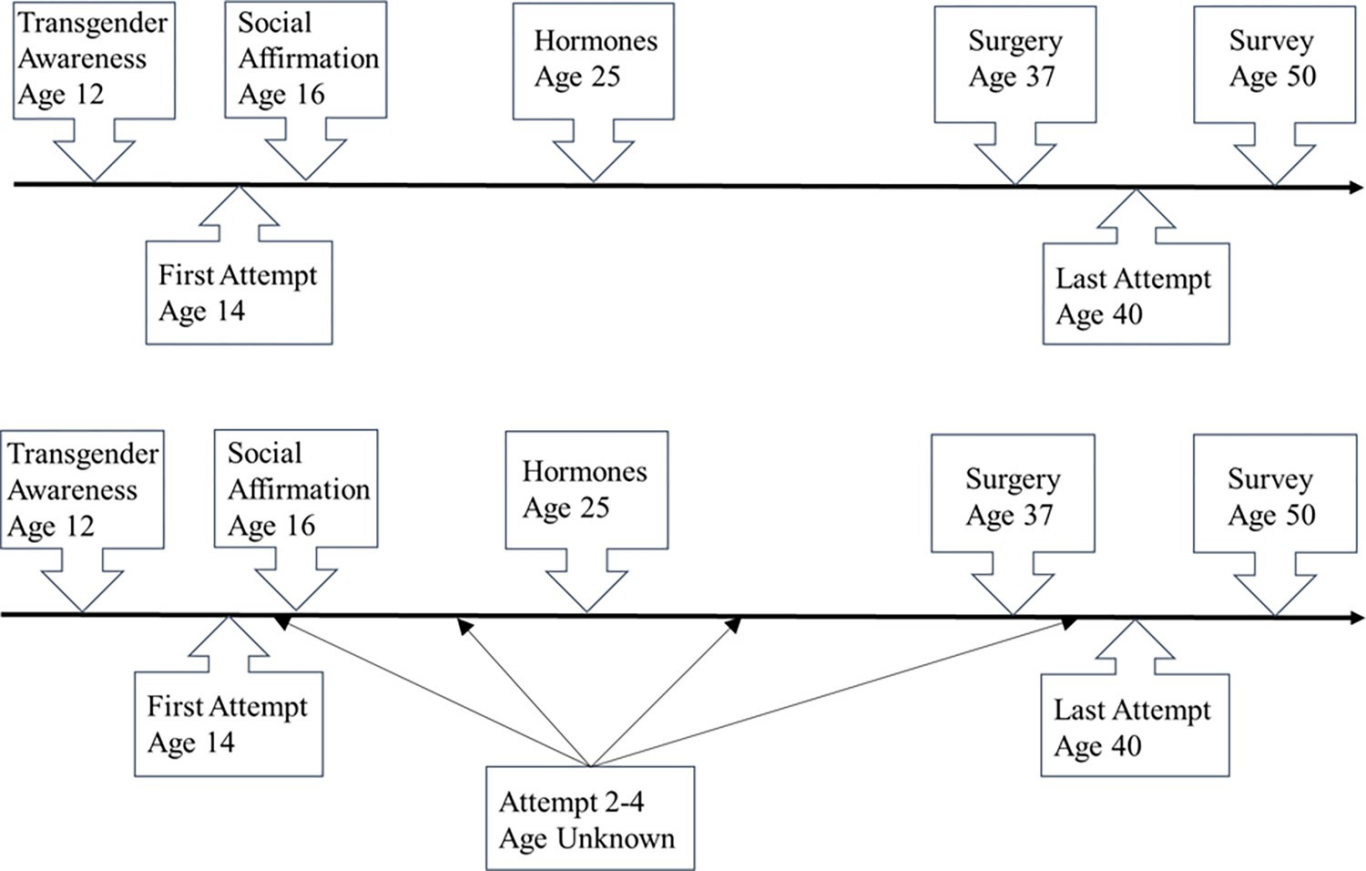
Two hypothetical participants showing complete information on exposures and events (a) and missing time of event (b). (a) Timeline example 1; (b) Timeline example 2.

The outcome of interest for our analysis is lifetime suicide attempts. Several variables were derived from the survey: 1) a binary indicator whether the participant had attempted suicide, 2) the number of attempts, 3) age at first attempt, and 4) age at last attempt. The total number of attempts was truncated at 26. If there were additional attempts between the first and last attempt, we do not know at what age they occurred. Consequently, recurrent-event survival analysis, which would otherwise be a natural choice for this type of data, becomes impossible to conduct directly. To illustrate the challenge in analyzing these data when age at suicide attempt is missing for some participants at some events, Fig 1 compares two participants having different numbers of attempts and ages. As shown, participant (a) had two attempts and no missing data because there are no attempts before the first and last attempts. Therefore, temporal relationship between attempts and gender affirmation is clear, making the assessment of the association between gender affirmation and suicide attempts relatively straightforward. By contrast, participant (b) had five suicide attempts and the age at the 2^nd^, 3^rd^, and 4^th^ attempt are unknown. The arrows show all possible timings of these events relative to exposures, which suggest different associations between exposures and outcomes.

### Statistical analysis

In order to analyze all the attempts and evaluate their temporality relative to exposures, we impute missing age at attempt using three approaches. For each imputation approach the analysis consists of three steps:

1. Generating imputed data sets
2. Analyzing each of the imputed data sets using recurrent event survival analysis
3. Combining the estimates of interest using Rubin’s rules [13].

The following sections provide details on each step.

### Imputation methods

The three approaches differ on how much the imputation depends on the observed data. By comparing results from these approaches, we can then evaluate the impact of the assumptions. First, we assume age at attempt follows a uniform distribution irrelevant to the observed data. Next, we allow a slightly stronger dependence on the observed data by assuming the distribution of attempts over age is the same in the observed and unobserved data. Last, we assume covariates also affect the distribution of age at attempt, in the same manner in the observed and unobserved data. Details on assumptions and implementations are discussed below.

First, a simple random imputation (denoted as SRI) approach assumes attempts randomly occurred between age at first attempt and age at last attempt following a uniform distribution (approach 1). An integer was draw with replacement from a uniform distribution ranging from age at first attempt to age at last attempt for each attempt with missing age.

Secondly, a modified SRI approach assumes attempts randomly occurred between age at first attempt and age at last attempt with probabilities of the observed values of age at attempt used to generate the missing data (approach 2). There are three steps to this approach. First, the probabilities of making suicide attempts at all ages were obtained from the observed dataset with each participant contributing up to two ages. Second, for each unique pair of age at first attempt and age at last attempt, probabilities within the range were standardized to add up to 1. Finally, an integer was drawn with replacement from a distribution using the probabilities from step 2 for each attempt with missing age. For each approach, 10 imputation data sets were generated.

The two approaches above assumed missing completely at random (MCAR) within age stratum while the data may actually be missing at random (MAR). To accommodate this possibility, a multiple imputation (MI) approach (approach 3) using linear regression method for continuous variable with univariate missing pattern was also explored. Data were transposed so that each row was an attempt and year of attempt is the only variable that could be missing. Age at attempt was assumed to be normally distributed and the imputed values were rounded to the nearest integers. Variables used to predict missing ages included age at first attempt, age at last attempt, age at survey, integer representing the order of attempt (2^nd^, 3^rd^, etc.), age at transgender awareness, race/ethnicity, sex, and maximum level of education. Note that age at survey can be treated as a baseline characteristic and included in the regression model because all the participants were surveyed at the same year and age at survey is equivalent to year of birth, which was prior to age at transgender awareness. To impose participant-specific upper bound (age at last attempt) and lower bound (age at first attempt), after each imputation, values out of range were re-set to missing and the imputation process was repeated until all values were within range (this required <100 iterations). Attempts of each participant were sorted by age and a new order of attempt was generated to replace the old. We also imputed on the log-transformed scale due to observed right-skewness and compared it to the original to assess the sensitivity of results to normality assumption. Here we rounded age to be consistent with the other two approaches; however, results are similar if we do not round age. We used the monotone regression method implemented by the standard SAS procedure PROC MI with MONOTONE REG. Details of this method was documented elsewhere [23, 24].

We implemented all three approaches and examined how the modelling results differed.

### Recurrent event survival analysis

After all ages of suicide attempts were imputed as above, the next step is to evaluate the association of time to suicide attempt and time-varying gender affirmation using recurrent event survival analysis. Modeling was carried out using a Cox proportional-hazards (PH) model and it is assumed that a) all the recurrent events are identical, i.e., the order of event is negligible and b) the PH assumption is satisfied for all variables. Unlike non-recurrent event models, this recurrent event model kept the participants in the risk set until the last attempt or censoring. The data were constructed so that each observation, or interval, corresponds to each recurrent event while gender affirmation remained unchanged. For participants with more than one interval, the different observations contributed by the same participants were treated as if they were independent. To adjust for the likely correlation among recurrent events on the same participant, robust variance estimates were used. All the analyses were conducted in SAS 9.4.

The model can be written as:

$$h(t,X(t))=h_{0}exp\left[\sum_{i=1}^{p1}\beta_{i}X_{i}+\sum_{j=1}^{p2}\delta_{j}X_{j}(t)\right]$$

Gender affirmation, represented by three variables including social affirmation, hormones, and surgery, was considered time-dependent, as denoted by the *X_j_*(*t*) variables. All the other variables, including gender, age at transgender awareness, race/ethnicity, and maximum level of education, were considered time-independent, as denoted by the *X_i_* variables. Maximum education acted as a proxy for social economic status of the participant. Thus, it was preferred over time-varying level of education, which was confounded by lapse of time since transgender awareness.

### Combining the estimates

The recurrent event survival analysis was conducted on each imputed dataset using the PROC PHREG procedure. Then parameter estimates and associated variance estimates were combined using the PROC MIANALYZE procedure to derive valid inferences for these parameters.

With m imputations, the combined point estimate of a parameter is the mean of the m individual estimates. By Rubin’s rule, the combined variance is:

$$T=\bar{W}+\left(1+\frac{1}{m}\right)B$$

Where $\bar{W}$ is the within-imputation variance of the variance estimates, and B is the between-imputation variance of the variance estimates.

Simulation studies show that this approach has reasonable coverage, bias and mean squared error (S4 Table).

## Results

The demographics are summarized in the initial report [11]. From this report, the study population is 84% white, 57% male assigned at birth and 31%, 18%, 20%, and 31% were 18–24, 25–29, 30–39, and > = 40 years of age, respectively, at survey completion. In addition, 35%, 25%, 19%, and 21% were < = 10, 11–14, 15–18 and >18 years of age, respectively, at awareness and 2%, 11%, 36%, 10%, 26%, and 15% of participants were in the following 6 education level categories: less than high school, high graduate, some college, associate’s degree, bachelor’s degree, and graduate/professional degree.

**Fig 2:**
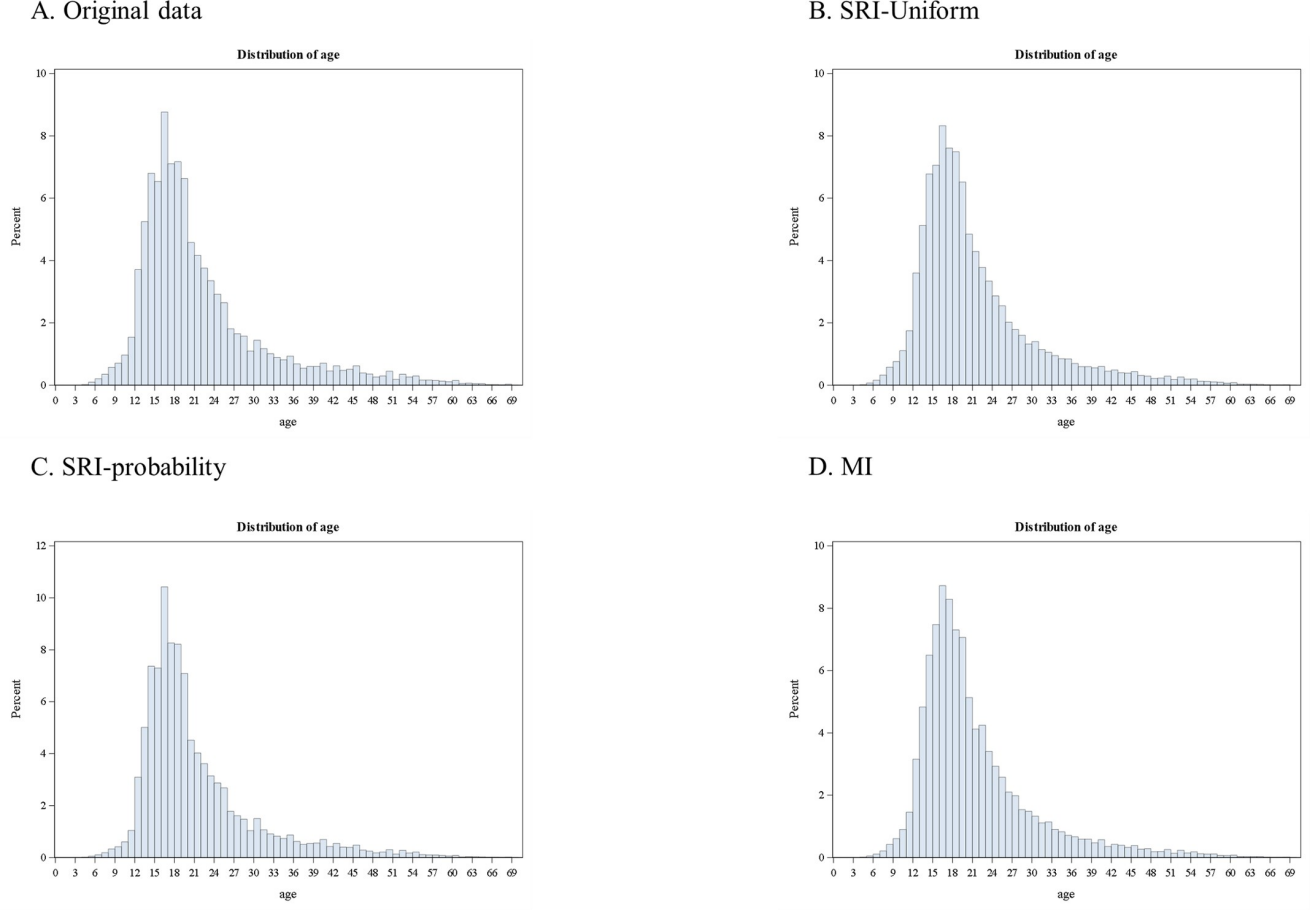
Histogram of original data (2A) and 10 datasets generated from three imputation approaches. SRI-uniform (2B, assumes attempts randomly occurred following a uniform distribution), SRI-probability (2C, assumes probabilities of the observed values of age at attempt used to generate the missing data) and MI (2D, multiple imputation regression model).

Histograms (Fig 2) show the distributions of age at attempts in the 10 imputed datasets combined compared to the original data. Imputed datasets all have the distribution of age at suicide attempt similar to that in the originally observed data.

**Fig 3:**
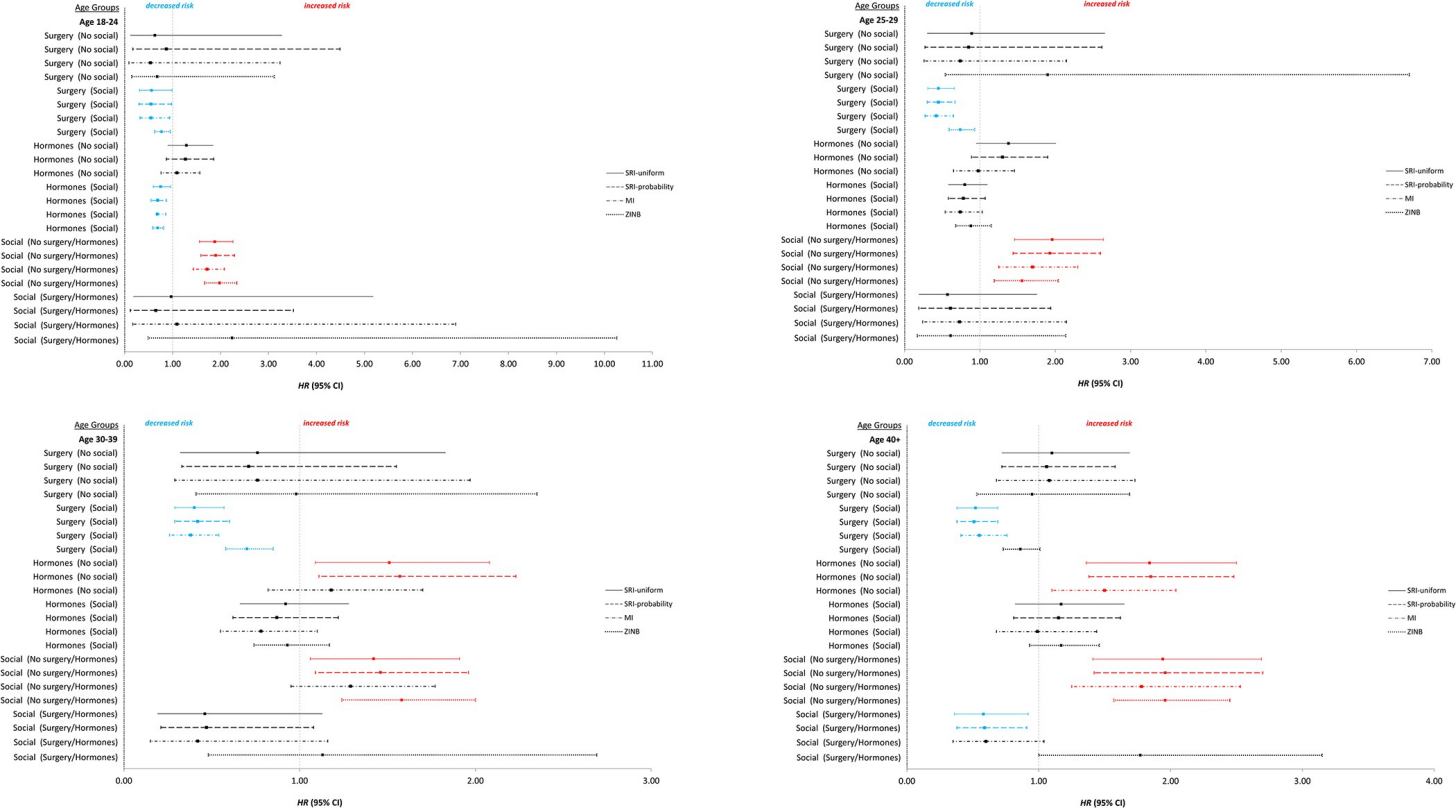
Similar hazard ratios for suicide for gender affirmation categories comparisons using the three imputation approaches by age group Age 18–24 (3A), Age 25–29 (3B), Age 30–39 (3C) and Age > = 40 (3D). Three imputation approaches are (1) SRI-uniform = assumes attempts randomly occurred following a uniform distribution, (2) SRI-probability assumes probabilities of the observed values of age at attempt used to generate the missing data, (3) MI uses multiple imputation regression model, and (4) zero-inflated negative binomial (ZINB) model used in the initial analysis. Reference group = no gender affirmation of any type. Social affirmation (No surgery/hormones) = participants had social affirmation but did not have surgery or hormones. Social affirmation (Surgery or hormones) = participants had social affirmation and either or both of surgery and hormones. Hormone-social affirmation interaction is not statistically significant thus not included in the ZINB model. Therefore, only the main effect of hormone is shown under Hormone (Social).

In the binary gender identity group, we found moderate associations of surgery and hormones on estimated hazard ratio (HR) as well as interactions with social affirmation that are differential by age. Table 1 shows the estimated unadjusted hazard ratio and the 95% confidence interval for each imputation approach. Table 2 and Fig 3 show the estimated adjusted HR and 95% confidence interval for each imputation approach. Surgery with social affirmation is consistently associated with decreased risk for suicide attempt across age groups, with small variation among imputation approaches (Table 2, HR = 0.55–0.56 in 18–24, 0.40–0.45 in 25–29, 0.38–0.42 in 30–39, and 0.51–0.55 in 40). Hormones with social affirmation is associated with decreased risk only in 18–24 group (HR 0.66–0.75); whereas in 30–39 and 40+ group, hormones without social affirmation is associated with increased risk (HR 1.51–1.85). Social affirmation without hormones or surgery is associated with increased risk using all imputation methods for 3 of the 4 age groups (HR = 1.65–1.90 in 18–24, 1.62–1.96 in 25–29 and 1.71–1.96 in 40+). Additionally, in 40+ group, social affirmation with hormones or surgery is associated with decreased risk only with the SRI approaches (HR = 0.58–0.59). Incidence rate ratios (IRRs) from the initial analysis using zero-inflated negative binomial model are also included for comparison.

**Table 1:** Unadjusted hazard ratios of gender affirmation in binary gender identity group compared among imputation methods. The HRs (IRRs for initial analysis) that are bolded represent those significantly different from one (p<0.05). Reference group = no gender affirmation of any type. Social (No surgery/hormones) = participants had social affirmation but did not have surgery or hormones. Social (Surgery or hormones) = participants had social affirmation and either or both of surgery and hormones.

		Imputation Approaches								Initial Analysis	
		SRI-uniform*		SRI-probability*		MI *		MI-log *		ZINB*	
Age at Survey	Gender Affirmation Groups	HR	95% CI	HR	95% CI	HR	95% CI	HR	95% CI	IRR	95% CI
Age 18–24	Surgery (No social)	0.39	(0.07–2.22)	0.54	(0.09–3.04)	0.31	(0.05–1.98)	0.35	(0.06–2.04)	0.76	(0.18–3.15)
	Surgery (Social)	**0.49**	**(0.27–0.89)**	**0.48**	**(0.26–0.89)**	**0.48**	**(0.27–0.85)**	**0.48**	**(0.27–0.88)**	**0.66**	**(0.55–0.79)**
	Hormones (No social)	1.19	(0.84–1.67)	1.21	(0.84–1.75)	1.15	(0.80–1.65)	1.05	(0.74–1.49)	NA**	
	Hormones (Social)	**0.64**	**(0.50–0.81)**	**0.60**	**(0.47–0.75)**	**0.60**	**(0.47–0.77)**	**0.58**	**(0.46–0.75)**	**0.67**	**(0.58–0.78)**
	Social (No surgery/hormones)	**1.95**	**(1.62–2.34)**	**2.01**	**(1.67–2.41)**	**1.93**	**(1.60–2.34)**	**1.84**	**(1.52–2.22)**	**1.96**	**(1.67–2.30)**
	Social (Surgery or hormones)	1.30	(0.22–7.58)	0.89	(0.15–5.27)	1.57	(0.23–10.61)	1.42	(0.25–8.24)	1.70	(0.41–7.09)
Age 25–29	Surgery (No social)	0.66	(0.22–1.97)	0.63	(0.21–1.93)	0.58	(0.20–1.65)	0.60	(0.21–1.74)	1.81	(0.52–6.34)
	Surgery (Social)	**0.38**	**(0.26–0.56)**	**0.38**	**(0.25–0.57)**	**0.36**	**(0.23–0.55)**	**0.34**	**(0.23–0.52)**	**0.61**	**(0.49–0.74)**
	Hormones (No social)	1.08	(0.74–1.56)	1.03	(0.71–1.50)	0.90	(0.61–1.33)	0.90	(0.61–1.35)	NA	
	Hormones (Social)	**0.66**	**(0.47–0.91)**	**0.64**	**(0.47–0.89)**	**0.67**	**(0.48–0.94)**	**0.66**	**(0.46–0.94)**	0.78	(0.61–1.00)
	Social (No surgery/hormones)	**1.75**	**(1.32–2.32)**	**1.75**	**(1.32–2.32)**	**1.64**	**(1.23–2.20)**	**1.56**	**(1.17–2.08)**	**1.67**	**(1.29–2.17)**
	Social (Surgery or hormones)	0.61	(0.20–1.88)	0.66	(0.21–2.06)	0.76	(0.26–2.23)	0.65	(0.22–1.89)	0.56	(0.16–1.95)
Age 30–39	Surgery (No social)	0.58	(0.25–1.37)	0.54	(0.25–1.15)	0.59	(0.23–1.52)	0.52	(0.22–1.23)	0.70	(0.30–1.63)
	Surgery (Social)	**0.30**	**(0.21–0.42)**	**0.31**	**(0.22–0.44)**	**0.29**	**(0.20–0.41)**	**0.30**	**(0.21–0.44)**	**0.52**	**(0.44–0.62)**
	Hormones (No social)	1.30	(0.95–1.77)	1.34	(0.96–1.88)	1.12	(0.79–1.59)	1.09	(0.78–1.53)	NA	
	Hormones (Social)	0.82	(0.59–1.15)	0.77	(0.55–1.09)	0.74	(0.52–1.06)	0.77	(0.54–1.10)	0.94	(0.75–1.17)
	Social (No surgery/hormones)	1.28	(0.96–1.71)	1.31	(0.97–1.75)	1.23	(0.90–1.67)	1.10	(0.82–1.47)	**1.55**	**(1.22–1.97)**
	Social (Surgery or hormones)	0.42	(0.17–1.03)	**0.43**	**(0.19–0.98)**	0.40	(0.14–1.09)	0.45	(0.18–1.14)	1.16	(0.50–2.67)
Age 40+	Surgery (No social)	1.02	(0.66–1.57)	0.98	(0.66–1.45)	1.02	(0.62–1.68)	0.93	(0.61–1.42)	0.88	(0.49–1.59)
	Surgery (Social)	**0.42**	**(0.31–0.56)**	**0.41**	**(0.31–0.55)**	**0.45**	**(0.33–0.62)**	**0.45**	**(0.33–0.61)**	**0.71**	**(0.60–0.84)**
	Hormones (No social)	**1.50**	**(1.12–2.00)**	**1.48**	**(1.12–1.95)**	**1.29**	**(0.95–1.74)**	**1.31**	**(0.98–1.76)**	NA	
	Hormones (Social)	0.99	(0.70–1.41)	0.97	(0.68–1.37)	0.86	(0.59–1.26)	0.91	(0.62–1.32)	1.02	(0.81–1.28)
	Social (No surgery/hormones)	**2.11**	**(1.54–2.88)**	**2.10**	**(1.55–2.86)**	**2.00**	**(1.42–2.83)**	**1.90**	**(1.36–2.67)**	**2.28**	**(1.82–2.86)**
	Social (Surgery or hormones)	**0.57**	**(0.36–0.91)**	**0.58**	**(0.38–0.90)**	0.60	(0.34–1.06)	0.63	(0.38–1.05)	**1.83**	**(1.02–3.29)**

*Abbreviations for multiple imputation type: SRI = simple random imputation; SRI-uniform = assumes attempts randomly occurred following a uniform distribution; SRI-probability = probabilities of the observed values of age at attempt used to generate the missing data; MI = multiple imputation regression model; ZINB = zero-inflated negative binomial model.

** Hormone-social affirmation interaction is not statistically significant thus not included in the ZINB model. Therefore, only the main effect of hormone is shown under Hormone (Social).

**Table 2:** Adjusted hazard ratios of gender affirmation in binary gender identity group compared among imputation methods. Model adjusts for age at transgender awareness, race/ethnicity, assigned sex at birth, and education. The HRs (IRRs for initial analysis) that are bolded represent those significantly different from one (p<0.05). Reference group = no gender affirmation of any type. Social (No surgery/hormones) = participants had social affirmation but did not have surgery or hormones. Social (Surgery or hormones) = participants had social affirmation and either or both of surgery and hormones.

		Imputation Approaches								Initial Analysis	
		SRI-uniform*		SRI-probability*		MI*		MI-log*		ZINB*	
Age at Survey	Gender Affirmation Groups	HR	95% CI	HR	95% CI	HR	95% CI	HR	95% CI	IRR	95% CI
Age 18–24	Surgery (No social)	0.63	(0.12–3.28)	0.87	(0.17–4.49)	0.54	(0.09–3.24)	0.59	(0.11–3.20)	0.68	(0.15–3.12)
	Surgery (Social)	**0.56**	**(0.31–0.99)**	**0.55**	**(0.30–0.98)**	**0.55**	**(0.32–0.94)**	**0.56**	**(0.32–0.97)**	**0.77**	**(0.63–0.95)**
	Hormones (No social)	1.29	(0.90–1.85)	1.27	(0.87–1.86)	1.09	(0.76–1.57)	1.01	(0.70–1.46)	NA**	
	Hormones (Social)	**0.75**	**(0.60–0.96)**	**0.69**	**(0.55–0.87)**	**0.68**	**(0.53–0.86)**	**0.66**	**(0.52–0.84)**	**0.69**	**(0.59–0.81)**
	Social (No surgery/hormones)	**1.88**	**(1.56–2.26)**	**1.90**	**(1.59–2.29)**	**1.72**	**(1.43–2.08)**	**1.65**	**(1.37–1.99)**	**1.98**	**(1.67–2.34)**
	Social (Surgery or hormones)	0.97	(0.18–5.18)	0.65	(0.12–3.52)	1.09	(0.17–6.90)	1.01	(0.19–5.32)	2.24	(0.49–10.26)
Age 25–29	Surgery (No social)	0.89	(0.30–2.66)	0.85	(0.27–2.62)	0.74	(0.26–2.15)	0.78	(0.27–2.26)	1.90	(0.54–6.70)
	Surgery (Social)	**0.45**	**(0.31–0.66)**	**0.45**	**(0.30–0.67)**	**0.42**	**(0.27–0.65)**	**0.40**	**(0.27–0.60)**	**0.74**	**(0.59–0.93)**
	Hormones (No social)	1.38	(0.95–2.01)	1.30	(0.89–1.90)	0.98	(0.65–1.46)	1.02	(0.68–1.54)	NA	
	Hormones (Social)	0.80	(0.58–1.10)	0.78	(0.58–1.07)	0.74	(0.54–1.03)	0.76	(0.54–1.06)	0.88	(0.68–1.15)
	Social (No surgery/hormones)	**1.96**	**(1.46–2.64)**	**1.93**	**(1.44–2.60)**	**1.70**	**(1.25–2.30)**	**1.62**	**(1.20–2.19)**	**1.56**	**(1.19–2.04)**
	Social (Surgery or hormones)	0.57	(0.19–1.76)	0.61	(0.19–1.94)	0.73	(0.24–2.15)	0.62	(0.21–1.82)	0.61	(0.17–2.14)
Age 30–39	Surgery (No social)	0.76	(0.32–1.83)	0.71	(0.33–1.55)	0.76	(0.29–1.97)	0.67	(0.28–1.61)	0.98	(0.41–2.35)
	Surgery (Social)	**0.40**	**(0.29–0.57)**	**0.42**	**(0.29–0.60)**	**0.38**	**(0.26–0.54)**	**0.40**	**(0.28–0.57)**	**0.70**	**(0.58–0.85)**
	Hormones (No social)	**1.51**	**(1.09–2.08)**	**1.57**	**(1.11–2.23)**	1.18	(0.82–1.70)	1.18	(0.83–1.69)	NA	
	Hormones (Social)	0.92	(0.66–1.28)	0.87	(0.62–1.22)	0.78	(0.55–1.10)	0.83	(0.59–1.18)	0.93	(0.74–1.17)
	Social (No surgery/hormones)	**1.42**	**(1.06–1.91)**	**1.46**	**(1.09–1.96)**	1.29	(0.95–1.77)	1.16	(0.87–1.56)	**1.58**	**(1.24–2.00)**
	Social (Surgery or hormones)	0.46	(0.19–1.13)	0.47	(0.21–1.08)	0.42	(0.15–1.16)	0.48	(0.19–1.22)	1.13	(0.48–2.69)
Age 40+	Surgery (No social)	1.10	(0.72–1.69)	1.06	(0.72–1.58)	1.08	(0.68–1.73)	0.99	(0.65–1.50)	0.95	(0.53–1.69)
	Surgery (Social)	**0.52**	**(0.38–0.69)**	**0.51**	**(0.38–0.69)**	**0.55**	**(0.41–0.76)**	**0.55**	**(0.40–0.76)**	0.86	(0.73–1.01)
	Hormones (No social)	**1.84**	**(1.36–2.50)**	**1.85**	**(1.38–2.48)**	**1.50**	**(1.10–2.04)**	**1.57**	**(1.15–2.13)**	NA	
	Hormones (Social)	1.17	(0.82–1.65)	1.15	(0.81–1.62)	0.99	(0.68–1.44)	1.05	(0.72–1.53)	1.17	(0.93–1.46)
	Social (No surgery/hormones)	**1.94**	**(1.41–2.69)**	**1.96**	**(1.42–2.70)**	**1.78**	**(1.25–2.53)**	**1.71**	**(1.21–2.42)**	**1.96**	**(1.57–2.45)**
	Social (Surgery or hormones)	**0.58**	**(0.36–0.92)**	**0.59**	**(0.38–0.91)**	0.60	(0.35–1.04)	0.64	(0.39–1.05)	1.77	(1.00–3.15)

*Abbreviations for multiple imputation type: SRI = simple random imputation; SRI-uniform = assumes attempts randomly occurred following a uniform distribution; SRI-probability = probabilities of the observed values of age at attempt used to generate the missing data; MI = multiple imputation regression model; ZINB = zero-inflated negative binomial model.

** Hormone-social affirmation interaction is not statistically significant thus not included in the ZINB model. Therefore, only the main effect of hormone is shown under Hormone (Social).

Simple random imputation assuming uniform distribution or using probabilities lead to similar point estimates and conclusions. The estimated HRs are consistent across imputation methods for most of the comparisons. However, there are a few comparisons within the 30–39 and 40+ age groups for which the estimated HR for the MI approach is closer to 1 relative to the estimated HR for the SRI approaches. These differences are detected when evaluating the association between hormones and suicide attempts for those without social affirmation and social affirmation for those with hormones but not surgery in the 30–39 year age group and when evaluating the social affirmation for those with hormone or surgery in those 40 years or older. The results are also similar for the MI on the original scale and the log scale. Within each imputation method, we generate 10 sample data sets. Similar point estimates and slightly narrower 95% confidence intervals were found when we generated 100 data sets (S1 Table).

## Discussion

A challenge faced in this analysis of gender affirmation and suicide risk among USTS transgender participants was data missing by design. When evaluating repeated time to event outcomes, it is ideal to use a recurrent survival time model. We were limited in this study due to the fact that the age of each suicide attempt was not collected. To address this challenge, we used three approaches to impute the age at suicide attempt and then fit a recurrent survival analysis with a time-varying covariate for gender affirmation.

Similar estimates and conclusions are found for the three imputation methods for the majority of the comparisons (87.5%, 21/24). We observed greater similarity between the two SRI approaches than between SRI approaches and MI. The main difference between the SRI and MI approaches is that for three comparisons the MI estimates were closer to the null hypothesis; however, the estimates are in the same direction.

Conclusions are consistent with previous survival analysis and analysis of attempt [12], showing negative associations between surgery and suicide attempts in participants who also had social affirmation in a binary gender identity population. But there are some differences between them. For example, in the analysis of attempt rate using zero-inflated negative binomial model, the rate ratios of having surgery versus not in participants who also had social affirmation ranged from 0.7 to 0.9 in participants aged 18–39 and was not statistically significant in participants aged 40 or above (RR 0.9, 0.7–1.0, p = 0.0712). In the recurrent survival analysis using the imputed data, however, the magnitude of association is larger, hazard ratios ranging from 0.4 to 0.6, and statistically significant across all age groups. The cause of the difference is likely to be that the older group was affected the most by lack of temporality in the analysis of attempt rate. This is because, in the zero-inflated negative binomial model, attempts that happened before surgery are lumped together with those that happened after surgery while only the latter is attributable to having surgery.

Another difference from the non-recurrent based analysis is that we additionally found an interaction between social affirmation and hormones which could resolve the contradictory findings on hormones in different age groups. In the zero-inflated negative binomial model [11], we found hormones associated with increased risk in 40+ group but decreased risk in 18–24 age group. However, analysis on imputed data revealed that social affirmation moderates this difference. Specifically, we found hormones without social affirmation associated with increased risk but hormone with social affirmation associated with decreased risk in 18–24 age group. These additional statistically significant findings are probably due to increased statistical power after missing data are imputed and included in the analysis.

We are aware that there are other imputation methods to consider, such as maximum likelihood, and multiple imputation using Cox models. Nevertheless, these alternative methods face some difficulties, including the necessity of specialized software and complex structure of the final analysis model. For instance, Paul Allison has listed several advantages of the multiple likelihood imputation method over MI, including more efficiency, fewer decisions involved, and lack of conflict between the imputation and the analysis model [14]. However, we faced some difficulty implementing the ML method with our data because the PHREG procedure in SAS, which is the procedure we used for all the other analyses, does not impute for missing data. And to our knowledge, there is no ready-to-use software that can impute failure time for recurrent event survival analysis. It is nevertheless an approach worthy of exploration in the future.

We also considered more complex models, such as mixture cure model allowing us to model the probability that participants are cured and will not have any attempts after gender affirmation. The Cox PH model we used assumes that all the recurrent events are identical. This is a commonly used and straightforward model but there are other modeling options that may more accurately reflect the nature of suicide attempts. For example, the accelerated failure time model is a parametric model that assumes the covariates increase or decrease survival time, instead of the hazard [25]. The mixture cure model jointly models a response variable for multiple groups with differential probabilities of susceptibility. This will allow us to assume that participants with certain characteristics or having had certain gender affirmation will be cured and never have any suicide attempts. Our previous use of zero-inflated negative binomial model on count data adopted a similar idea. With the failure time data imputed, all these other modeling options can be explored in the future.

Finally, we recognize that external evidence could inform assumptions for imputation, but such evidence is very limited in this less researched population. Suicide attempt rates by age are available for the general U.S. population [26] but not for transgender population specifically [27]. Yet, suicide patterns may differ greatly between the two groups. It has been reported that the transgender population has a much higher suicide attempt rate than the general population [2]. It is highly likely that the distribution of suicide attempt also differs in these two populations. Therefore, the resultant limitation is that we cannot directly use data in general population to correct for the uncertainty in the suicide probabilities we used for imputation. The USTS survey is the first to provide data on age-specific suicide attempt rates in the transgender population. As more data are gathered, they should be utilized in imputation.

Findings have implications for future collection of suicide attempt data in the transgender population. Optimal suicide attempt measures need to both minimize potential recall bias and capture information of sufficient complexity and completeness to enable the implementation of ideal analytic methods. Future research may consider alternative designs to the cross-sectional survey to potentially reduce the bias. Participant-report measures of suicide attempt history are sensitive questions; thus, care must be taken to balance participant burden and potential upset. Research to validate and compare measures that ask first and last attempt to those which enumerate all attempts in this population will be important for future surveys.

## Conclusion

In conclusion, we evaluated three imputation approaches in addressing missing failure times in recurrent-event type of data. In our USTS data of suicide attempts and gender affirmation, parameter estimates are insensitive to imputation approaches. Findings from imputation methods are generally consistent with those from single-event survival analysis and zero-inflated negative binomial model. However, using the imputation approaches, we were able to identify additional associations that were not detected in the zero-inflated negative binomial model and were consistent with clinical expectations. Specifically, we found a statistically significant association between surgery and suicide attempts in 40+ group and an interaction between hormones and social affirmation in 18–24 group, while the zero-inflated negative binomial model failed to do so. These additional findings are likely due to increased statistical power and re-established temporality in the imputed data. Using the imputation approaches, we were able to confirm the findings and identify additional associations that were not detected in the initial analysis using the zero-inflated negative binomial model, highlighting the potential value of using imputation methods in the setting of missing failure times. Findings suggest the need to consider additional analytical approaches in settings with high levels of data missing by design and to conduct further measure validation for future surveys of suicide risk in the transgender population.

## Supporting information

S1 Fig Histogram of imputed age by approach (100 imputation datasets). (DOCX)

S1 Table Unadjusted hazard ratios of gender affirmation in binary gender identity group compared among imputation methods with 100 imputation datasets. Reference group = no gender affirmation of any type. Social (No surgery/hormones) = participants had social affirmation but did not have surgery or hormones. Social (Surgery or hormones) = participants had social affirmation and either or both of surgery and hormones.
(DOCX)

S2 Table Adjusted hazard ratios of gender affirmation in binary gender identity group compared among imputation methods with 100 imputation datasets. Reference group = no gender affirmation of any type. Social (No surgery/hormones) = participants had social affirmation but did not have surgery or hormones. Social (Surgery or hormones) = participants had social affirmation and either or both of surgery and hormones.
(DOCX)

S3 Table Variables used in the analysis. (DOCX)

S4 Table Simulation results evaluating the coverage, bias and mean squared error. (DOCX)
