# Supplementary material for: Imputation methods for missing failure times in recurrent-event survival analysis: Application to suicide attempts in the transgender population
Source: PLoS One. 2022 Dec 9;17(12):e0278913. doi: 10.1371/journal.pone.0278913 (PMC9733870; doi:10.1371/journal.pone.0278913)
Supplement: S1 Fig — (DOCX) [file pone.0278913.s001.docx]

Supplemental Figure 1. Histogram of imputed age by approach (100 imputation datasets)

B. SRI-probability

A. SRI-Uniform

C. MI
