# Supplementary material for: Imputation methods for missing failure times in recurrent-event survival analysis: Application to suicide attempts in the transgender population
Source: PLoS One. 2022 Dec 9;17(12):e0278913. doi: 10.1371/journal.pone.0278913 (PMC9733870; doi:10.1371/journal.pone.0278913)
Supplement: S1 Table — Reference group = no gender affirmation of any type. Social (No surgery/hormones) = participants had social affirmation but did not have surgery or hormones. Social (Surgery or hormones) = participants had social affirmation and either or both of surgery and hormones. (DOCX) [file pone.0278913.s002.docx]

Supplemental Table 1. **Unadjusted** hazard ratios of gender affirmation in **binary** gender identity group compared among imputation methods with 100 imputation datasets. Reference group=no gender affirmation of any type. Social (No surgery/hormones) = participants had social affirmation but did not have surgery or hormones. Social (Surgery or hormones) = participants had social affirmation and either or both of surgery and hormones.

| Method | SRI-uniform | | SRI-probability | |
| --- | --- | --- | --- | --- |
|  | HR | 95% CI | HR | 95% CI |
| **Age 18-24** |  |  |  |  |
| Surgery (No social) | 0.39 | (0.06-2.44) | 0.47 | (0.08-2.84) |
| Surgery (Social) | **0.50** | **(0.28-0.89)** | **0.48** | **(0.26-0.88)** |
| Hormones (No social) | 1.20 | (0.85-1.68) | 1.21 | (0.84-1.75) |
| Hormones (Social) | **0.64** | **(0.51-0.81)** | **0.60** | **(0.48-0.76)** |
| Social (No surgery/hormones) | **1.96** | **(1.63-2.36)** | **1.99** | **(1.65-2.39)** |
| Social (Surgery or hormones) | 1.35 | (0.21-8.71) | 1.01 | (0.16-6.27) |
| **Age 25-29** |  |  |  |  |
| Surgery (No social) | 0.64 | (0.22-1.90) | 0.60 | (0.20-1.75) |
| Surgery (Social) | **0.38** | **(0.26-0.57)** | **0.37** | **(0.25-0.57)** |
| Hormones (No social) | 1.06 | (0.73-1.52) | 1.05 | (0.73-1.51) |
| Hormones (Social) | **0.66** | **(0.47-0.91)** | **0.64** | **(0.47-0.88)** |
| Social (No surgery/hormones) | **1.76** | **(1.33-2.32)** | **1.76** | **(1.33-2.33)** |
| Social (Surgery or hormones) | 0.66 | (0.22-2.00) | 0.68 | (0.22-2.06) |
| **Age 30-39** |  |  |  |  |
| Surgery (No social) | 0.59 | (0.25-1.40) | 0.58 | (0.25-1.35) |
| Surgery (Social) | **0.30** | **(0.21-0.43)** | **0.31** | **(0.22-0.44)** |
| Hormones (No social) | 1.32 | (0.96-1.82) | 1.33 | (0.97-1.83) |
| Hormones (Social) | 0.82 | (0.58-1.15) | 0.79 | (0.56-1.11) |
| Social (No surgery/hormones) | 1.28 | (0.96-1.72) | 1.29 | (0.96-1.74) |
| Social (Surgery or hormones) | 0.41 | (0.16-1.00) | 0.41 | (0.17-1.00) |
| **Age 40+** |  |  |  |  |
| Surgery (No social) | 1.00 | (0.65-1.53) | 0.99 | (0.66-1.50) |
| Surgery (Social) | **0.42** | **(0.32-0.57)** | **0.42** | **(0.32-0.56)** |
| Hormones (No social) | **1.51** | **(1.14-2.01)** | **1.48** | **(1.12-1.96)** |
| Hormones (Social) | 0.97 | (0.68-1.38) | 0.98 | (0.69-1.39) |
| Social (No surgery/hormones) | **2.13** | **(1.56-2.91)** | **2.07** | **(1.52-2.81)** |
| Social (Surgery or hormones) | **0.58** | **(0.36-0.94)** | **0.58** | **(0.37-0.91)** |
