# Supplementary material for: Imputation methods for missing failure times in recurrent-event survival analysis: Application to suicide attempts in the transgender population
Source: PLoS One. 2022 Dec 9;17(12):e0278913. doi: 10.1371/journal.pone.0278913 (PMC9733870; doi:10.1371/journal.pone.0278913)
Supplement: S2 Table — Reference group = no gender affirmation of any type. Social (No surgery/hormones) = participants had social affirmation but did not have surgery or hormones. Social (Surgery or hormones) = participants had social affirmation and either or both of surgery and hormones. (DOCX) [file pone.0278913.s003.docx]

Supplemental Table 2**. Adjusted** hazard ratios of gender affirmation in **binary** gender identity group compared among imputation methods with 100 imputation datasets. Reference group=no gender affirmation of any type. Social (No surgery/hormones) = participants had social affirmation but did not have surgery or hormones. Social (Surgery or hormones) = participants had social affirmation and either or both of surgery and hormones.

| Method | SRI-uniform | | SRI-probability | |
| --- | --- | --- | --- | --- |
|  | HR | 95% CI | HR | 95% CI |
| Age 18-24 |  |  |  |  |
| Surgery (No social) | 0.62 | (0.11-3.65) | 0.76 | (0.14-4.22) |
| Surgery (Social) | **0.56** | **(0.32-0.99)** | **0.54** | **(0.30-0.97)** |
| Hormones (No social) | 1.30 | (0.91-1.86) | 1.27 | (0.86-1.86) |
| Hormones (Social) | **0.76** | **(0.60-0.96)** | **0.70** | **(0.56-0.89)** |
| Social (No surgery/hormones) | **1.89** | **(1.57-2.28)** | **1.88** | **(1.57-2.27)** |
| Social (Surgery or hormones) | 1.00 | (0.17-6.00) | 0.74 | (0.13-4.20) |
| Age 25-29 |  |  |  |  |
| Surgery (No social) | 0.86 | (0.29-2.57) | 0.80 | (0.27-2.37) |
| Surgery (Social) | **0.45** | **(0.30-0.67)** | **0.44** | **(0.29-0.66)** |
| Hormones (No social) | 1.35 | (0.93-1.96) | 1.31 | (0.90-1.91) |
| Hormones (Social) | 0.80 | (0.58-1.10) | 0.78 | (0.57-1.06) |
| Social (No surgery/hormones) | **1.97** | **(1.47-2.64)** | **1.94** | **(1.45-2.62)** |
| Social (Surgery or hormones) | 0.61 | (0.20-1.88) | 0.64 | (0.21-1.94) |
| Age 30-39 |  |  |  |  |
| Surgery (No social) | 0.86 | (0.29-2.57) | 0.76 | (0.32-1.82) |
| Surgery (Social) | **0.45** | **(0.30-0.67)** | **0.42** | **(0.29-0.59)** |
| Hormones (No social) | 1.35 | (0.93-1.96) | **1.56** | **(1.12-2.18)** |
| Hormones (Social) | 0.80 | (0.58-1.10) | 0.89 | (0.63-1.24) |
| Social (No surgery/hormones) | **1.97** | **(1.47-2.64)** | **1.44** | **(1.07-1.95)** |
| Social (Surgery or hormones) | 0.61 | (0.20-1.88) | 0.45 | (0.18-1.10) |
| Age 40+ |  |  |  |  |
| Surgery (No social) | 1.07 | (0.70-1.64) | 1.08 | (0.71-1.63) |
| Surgery (Social) | **0.52** | **(0.39-0.71)** | **0.52** | **(0.39-0.70)** |
| Hormones (No social) | **1.87** | **(1.39-2.51)** | **1.85** | **(1.38-2.48)** |
| Hormones (Social) | 1.14 | (0.80-1.62) | 1.16 | (0.82-1.64) |
| Social (No surgery/hormones) | **1.97** | **(1.43-2.71)** | **1.92** | **(1.40-2.64)** |
| Social (Surgery or hormones) | **0.59** | **(0.36-0.94)** | **0.58** | **(0.37-0.92)** |

* Adjusted for age at transgender awareness, race/ethnicity, assigned sex at birth, and education
