# Supplementary material for: Imputation methods for missing failure times in recurrent-event survival analysis: Application to suicide attempts in the transgender population
Source: PLoS One. 2022 Dec 9;17(12):e0278913. doi: 10.1371/journal.pone.0278913 (PMC9733870; doi:10.1371/journal.pone.0278913)
Supplement: S3 Table — (DOCX) [file pone.0278913.s004.docx]

Supplemental Table 3. Variables used in the analysis

| Variable | Survey Question | Categorization |
| --- | --- | --- |
| Number of suicide attempts | How many times have you tried to kill yourself in your lifetime? | Numeric, truncated at 26 |
| Age at attempt (if only one attempt) | How old were you when you tried to kill yourself? | Numeric |
| Age at first attempt (if more than one attempt) | How old were you the first time you tried to kill yourself? | Numeric |
| Age at last attempt (if more than one attempt) | How old were you the last time you tried to kill yourself? | Numeric |
| Current age | What is your current age? | 18-24  25-29  30-39  40 or above |
| Age at awareness | At about what age did you start to think you were trans (even if you did not know the word for it)? | 10 or below  11-14  15-18  Above 18 |
| Age at social affirmation | How old were you when you started to live full-time in a gender that is different from the one assigned to you at birth? | Numeric |
| Age beginning hormone | At what age did you begin hormone treatment/HRT treatment? | Numeric |
| Age at first surgery | At what age did you have your first procedure (top/chest surgery, hysterectomy, metoidioplasty, phalloplasty, or others)? | Numeric |
| Race/ Ethnicity | Race and ethnicity collapsed | Alaska Native/American Indian alone  Asian/NH/PI  Biracial/Multiracial/Not listed  Black/African American alone  Latino/a/Hispanic alone  White/ME/NA alone |
| Assigned sex at birth | What sex were you assigned at birth, on your original birth certificate? | Female  Male |
| Education | What is the highest level of school or degree you have completed? | Less than high school  High school grad (including GED)  Some college (no degree)  Associate’s Degree  Bachelor’s Degree  Graduate or professional degree |
