# Supplementary material for: Imputation methods for missing failure times in recurrent-event survival analysis: Application to suicide attempts in the transgender population
Source: PLoS One. 2022 Dec 9;17(12):e0278913. doi: 10.1371/journal.pone.0278913 (PMC9733870; doi:10.1371/journal.pone.0278913)
Supplement: S4 Table — (DOCX) [file pone.0278913.s005.docx]

Supplemental Table 4. Simulation results (n=2000). In this simulation, we first generated n=2000 simulation datasets from an underlying true recurrent survival model (true parameters are -0.3 for treatment terms, -0.02 for age at awareness, and -0.01 for current age). For each data set, we removed the data between the first and last suicide attempt, applied multiple imputation to generate 5 imputation datasets and implemented the recurrent event survival analysis by age group as we did in the paper. We evaluated the pooled parameter estimates against the true parameter in terms of coverage, bias, and mean standard error. Results below show that multiple imputation performs well in this setting with 92-95% coverage of the underlying true parameters.

| Term | Coverage | Bias | MSE |
| --- | --- | --- | --- |
| Social affirmation | 0.941 | -0.008074 | 0.008919 |
| Hormones | 0.936 | -0.031647 | 0.013622 |
| Surgery | 0.947 | -0.011484 | 0.031573 |
| Age at awareness | 0.928 | 0.001956 | 0.000029 |
